# Unraveling a Small Secreted Peptide SUBPEP3 That Positively Regulates Salt-Stress Tolerance in *Pyrus betulifolia*

**DOI:** 10.3390/ijms25094612

**Published:** 2024-04-23

**Authors:** Chaoran Xu, Ling Xiang, Wenting Huang, Xiao Zhang, Chong Mao, Shuang Wu, Tianzhong Li, Shengyuan Wang, Shengnan Wang

**Affiliations:** College of Horticulture, China Agricultural University, Beijing 100080, China

**Keywords:** small secreted peptides, *Pyrus betulifolia*, genome annotation, salt tolerance, subtilase peptide 3

## Abstract

Small secreted peptides (SSPs) play important roles in regulating plants’ growth and development in response to external stimulus, but the genes and functions of SSPs in many species are still unknown. Therefore, it is particularly significant to characterize and annotate SSP genes in plant genomes. As a widely used stock of pears, *Pyrus betulifolia* has strong resistance to biotic and abiotic stresses. In this study, we analyzed the SSPs genes in the genome of *P. betulifolia* according to their characteristics and homology. A total of 1195 SSP genes were identified, and most of them are signaling molecules. Among these, we identified a new SSP, subtilase peptide 3 (SUBPEP3), which derived from the PA region of preSUBPEP3, increasing the expression level under salt stress. Both adding synthetic peptide SUBPEP3 to the culture medium of pears and the overexpression of SUBPEP3 in tobacco can improve the salt tolerance of plants. In summary, we annotated the SSP genes in the *P. betulifolia* genome and identified a small secreted peptide SUBPEP3 that regulates the salt tolerance of *P. betulifolia*, which provides an important theoretical basis for further revealing the function of SSPs.

## 1. Introduction

Pears are a widely grown cash crop. In the process of pear seedling propagation, grafting is the most commonly used method of vegetative propagation. Grafting propagation plays an important role in maintaining the trait stability of scion varieties, shortening the juvenile phase, enhancing stress resistance, and regulating tree architecture [1]. Therefore, the application of excellent stocks is of great significance for improving fruit yield and quality and increasing economic benefits. Among them, *P. betulifolia* is a kind of stock that is widely used in pear seedling propagation. The herbarium of *Pyrus betulifolia* (BJM 155933) was deposited at the national natural history museum of China. It has the characteristics of good grafting affinity, a developed root system, and good drought tolerance, salt tolerance, cold tolerance, potassium (K) deficiency tolerance, and pear anthracnose resistance [2,3,4,5,6].

Salt stress can lead to water deficiency, ion toxicity, and the accumulation of reactive oxygen species (ROS) and other harmful substances, which seriously affect the normal growth and development of plants [7]. Soil salinization is an important global agricultural problem, which seriously affects agricultural productivity and environmental sustainability [8]. At present, soil improvement is carried out through freshwater washing and chemical application, but such methods consume a lot of manpower and material resources with little effect [9]. Therefore, it is particularly significant to breed excellent crop varieties with salt tolerance. During the cultivation of fruit trees, stocks with good salt tolerance are often used to improve the salt tolerance of seedlings [9].

The regulation of salt tolerance mechanisms is closely related to different signaling pathways and transcription factors. Various signaling pathways in response to salt stress include ROS signaling, Ca^2+^ signaling, plant hormone signaling, and some small signaling molecules. These signaling molecules can regulate downstream genes to control ion homeostasis and osmotic tolerance [10,11,12]. At present, with the deepening of research on SSPs, the role of SSPs in plant stress response and signal transduction is attracting more and more attention, and they are even being referred to as “peptide hormones” [13]. The common action mechanism of SSPs is to act as short- or long-distance signaling molecules and bind to the leucine-rich repeat receptor kinases (LRR-RKs) on the cell membrane, thereby causing a series of downstream cascades [14]. For example, N-deficient roots in *Arabidopsis* produce C-terminally encoded peptide 1 (CEP1), which moves long-distance to the shoots to bind to two LRR-RKs, thereby regulating downstream gene expression to enhance the compensatory uptake of nitrate by N-sufficient roots [15]. The CLAVATA3/EMBRYO SURROUNDING REGION-RELATED 25 (CLE25) peptide can respond to signals of water deficiency in roots and move to leaves over long distances. It binds to BARELY ANY MERISTEM (BAM) receptors to regulate abscisic acid biosynthesis and stomatal movements, thereby enhancing the drought resistance by regulating transpiration [16].

SSPs are usually 5–75 amino acids long and are derived from precursor proteins [14,17]. Previous studies have shown that they usually have several characteristics: the precursor proteins are relatively small (often <250 amino acids), have N-terminal signal peptides, and have no transmembrane domains [18]. Due to the above characteristics and the high conservation of SSPs, hidden Markov models (HMMs) have been used to predict the potential SSPs in the genomes of some species. *Medicago truncatula* Small Secreted Peptide Database (MtSSPdb) predicts 4439 SSP genes in the leguminous plants *Medicago truncatula* [18]. The PlantSSP database contains SSP genes in the genomes of 32 species [19]. Since SSPs often have post-translational modifications [20], bioinformatics prediction and nano-liquid chromatography–tandem mass spectrometry (LC-MS/MS)-based methods for detecting small peptides are jointly used to determine the sequence of SSPs [21].

In this study, we predicted the precursor proteins and functions of SSPs in the genome of *P. betulifolia* and screened a gene that responded to salt stress. Then, we demonstrated that the small secreted peptide SUBPEP3 could improve the salt tolerance of plants by applying synthetic peptides and overexpressing the SUBPEP3 gene in plants. These results provided a comprehensive analysis of SSPs in *P. betulifolia* and identified a peptide SUBPEP3 that regulates salt tolerance. It is of great significance to further explore the role of SSPs in plant salt tolerance and to breed excellent stock varieties.

## 2. Results

### 2.1. Analysis of SSP Genes in the Pyrus betulifolia Genome

**Figure 1:**
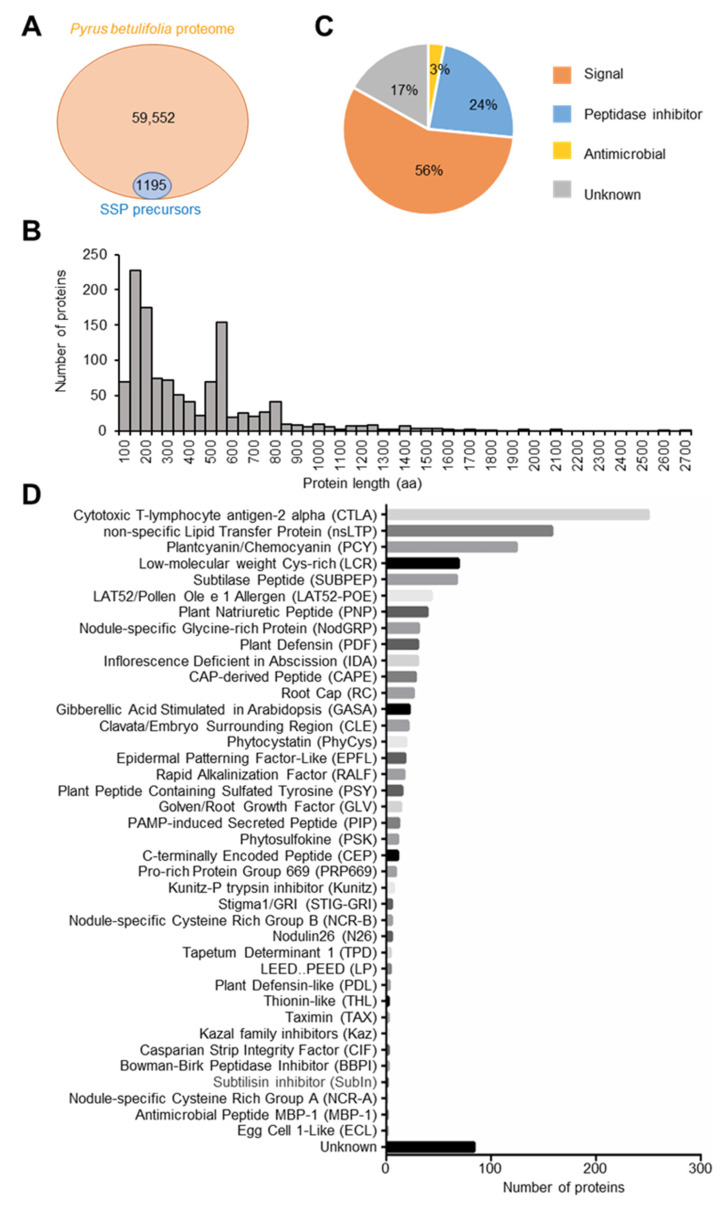
Identification of small secreted peptides (SSPs) in the *Pyrus betulifolia* protein database. (**A**) The number of SSPs identified in the *P. betulifolia* protein database. (**B**) The length of SSP precursor proteins identified in the *P. betulifolia* protein database. (**C**) The functional prediction of SSPs in *P. betulifolia*. *n* = 1195. (**D**) The number of proteins in the SSPs protein families in *P. betulifolia*. Different colors means different protein families.

**Figure 2:**
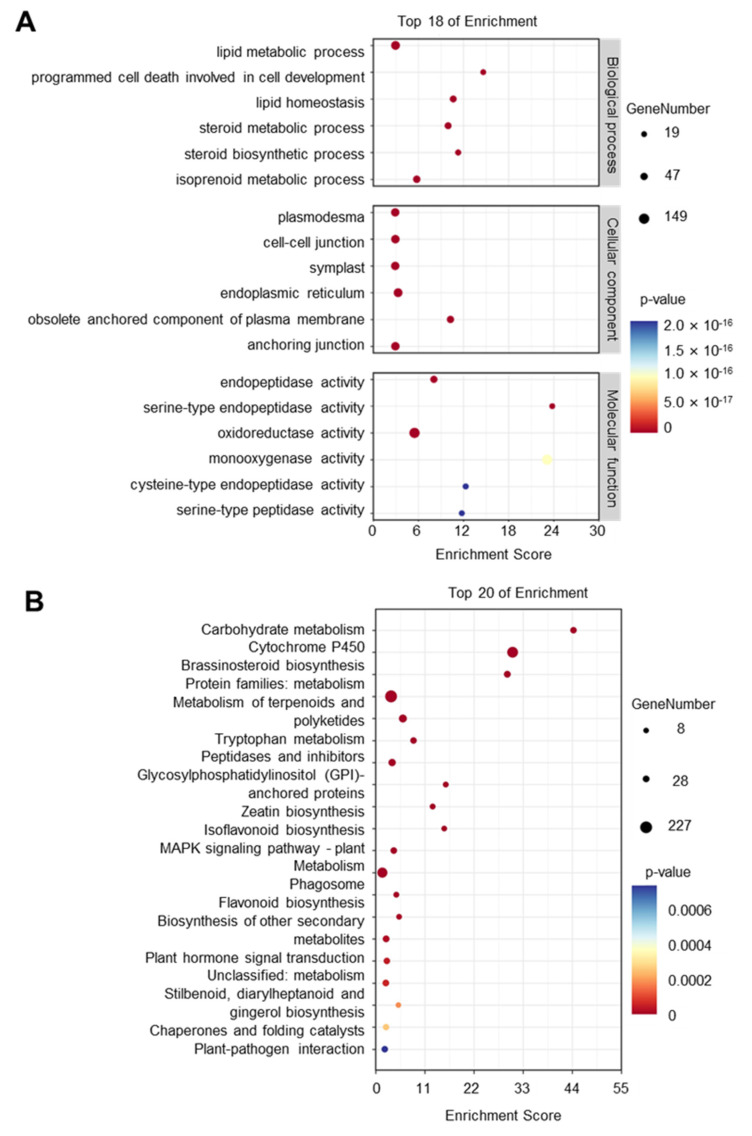
The function prediction of SSPs in *P. betulifolia.* (**A**) Gene ontology (GO) enrichment analysis results of SSPs in *P. betulifolia* were classified according to the biological process, cellular component, and molecular function. The top 18 GO term items with the minimum *p*-value and the most significant enrichment in each GO category are shown. (**B**) Kyoto Encyclopedia of Genes and Genomes (KEGG) enrichment analysis of SSPs in *P. betulifolia* shows the enriched items in the top 20.

There are 59,552 proteins according to the *P. betulifolia* protein database. Combined with the characteristics of SSPs and the homology of known SSPs, 1195 SSP precursor genes were identified by bioinformatics analysis (Figure 1A and Table S1). Counting the number of amino acids in these SSP precursor proteins, we found 46% of them were less than 250 aa (Figure 1B). The functions of SSPs were classified into three categories, including signaling molecules, peptidase inhibitors, and antimicrobial agents, and more than 50% of SSPs belonged to signaling molecules (Figure 1C). Based on the homology to known SSP protein families, we predicted the protein families of SSP genes. The top five gene families which have more genes than other identified SSP families are Cytotoxic T-lymphocyte antigen-2 alpha (CTLA), and non-specific Lipid Transfer Protein (nsLTP), Plantcyanin/Chemocyanin (PCY), low-molecular-weight Cys-rich (LCR), and Subtilase Peptide (SUBPEP) (Figure 1D). Gene ontology (GO) and the Kyoto Encyclopedia of Genes and Genomes (KEGG) enrichment analysis of the identified SSP genes showed that the degree of enrichment of the serine-type endopeptidase was the highest in GO enrichment analysis. This GO term corresponds to the gene family SUBPEP, which has been found to be related to plant defense response mechanisms (Figure 2A,B and Table S2). These results indicate that there are a large number of small peptides in *P. betulifolia* which are involved in various functions of growth and development.

### 2.2. The Characteristic of SUBPEP3

**Figure 3:**
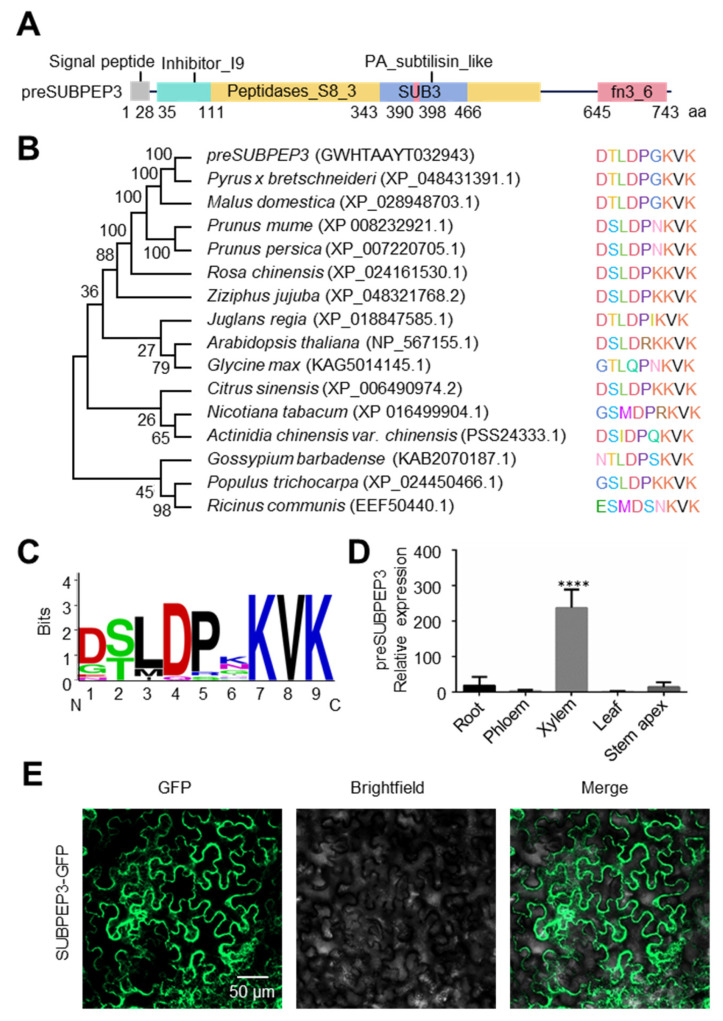
The characteristic of preSUBPEP3 and SUBPEP3. (**A**) Conserved domain of preSUBPEP3. The numbers represent amino acid positions. (**B**) Phylogenetic tree shows the clustering analysis of *preSUBPEP3* with homologous sequences from other plants. Alignment of the SUBPEP3 domains can be seen next to the phylogenetic tree. Different colors means different amino acids. (**C**) The Weblogo plot shows the SUBPEP3 domain conservation. (**D**) Expression pattern of *preSUBPEP3* in different tissues of *P. betulifolia*. Data are plotted as means ± SD and are based on three biological replicates. **** *p* < 0.0001 by Student’s *t*-test. (**E**) Subcellular localization of SUBPEP3-GFP.

In all the annotated small peptides, subtilase peptide 3 (SUBPEP3) was detected in our previous studies (unpublished data). It is a small secreted peptide that belongs to the SUBPEP family. preSUBPEP3, which belongs to a subtilisin-like protease, is the precursor protein of the small peptide SUBPEP3. Analysis of protein-conserved domains found that it has conserved domains common to plant subtilisin, including a signal peptide domain, a proprotein region (Inhibitor-I9), a peptidase S8 region (S8-peptide), a protease-associated domain (PA), and a C-terminal fibronectin III domain (FnIII) (Figure 3A). Among them, the small peptide SUBPEP3 is located in the PA region from 390 aa to 398 aa. Phylogenetic analysis showed that preSUBPEP3 is generally conserved among species of dicots (Figure 3B). The statistical analysis of its conserved amino acid sites showed that the seventh lysine, the eighth valine, and the ninth lysine were unchanged, and these amino sites may play an important role in the function of SUBPEP3 (Figure 3C). Tissue-specific analysis of preSUBPEP3 revealed that its expression was significantly higher in the xylem than in the root, phloem, leaf, and shoot (Figure 3D). Subcellular localization analysis showed that SUBPEP3 were localized in the cytoplasm and cell membrane (Figure 3E). In conclusion, SUBPEP3 is a small peptide that is conserved among different species and may play a role in regulating plant growth and development through long-distance transport.

### 2.3. preSUBPEP3 Response to Salt Stress

**Figure 4:**
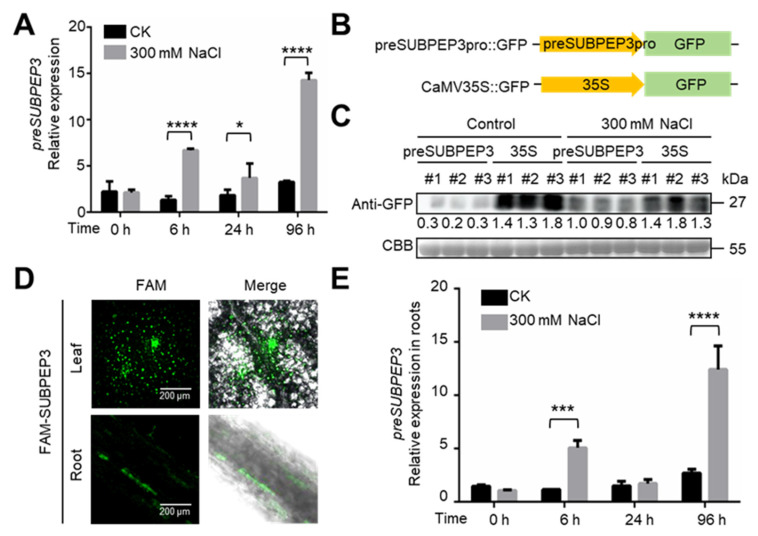
The expression pattern of preSUBPEP3 under salt stress. (**A**) Relative expression of *preSUBPEP3* of *P. betulifolia* tissue culture seedling at 0, 6, 24, and 96 h after salt treatment. (**B**) The schematic diagram shows the vectors overexpressing green fluorescent protein (GFP); the promoter is the promoter of *preSUBPEP3* or *CaMV35S*. preSUBPEP3pro is the promoter of *preSUBPEP3*. (**C**) Detection of the amount of GFP proteins in infiltrated *P. betulifolia* which were treated with 300 mM Nacl or not. Western blot was performed by anti-GFP antibody. CBB—total protein staining by Coomassie brilliant blue. #1, #2, and #3 refer to three independently infiltrated plants. (**D**) The fluorescence images of the leaves and roots at 3 d after *P. betulifolia* culture seedlings cultured in the medium with 10 µM FAM-SUBPEP3. A scale bar is shown in each subfigure. (**E**) Relative expression of *preSUBPEP3* in the roots of *P. betulifolia* tissue culture seedlings at 0, 6, 24, and 96 h after salt treatment. Data are plotted as means ± SD and are based on three biological replicates. * *p* < 0.05 by Student’s *t*-test. *** *p* < 0.001 by Student’s *t*-test. **** *p* < 0.0001 by Student’s *t*-test.

To explore the ability of preSUBPEP3 to resist abiotic stress in *P. betulifolia*, we treated *P. betulifolia* tissue culture seedlings with 300 mM NaCl and detected the expression of *preSUBPEP3* at 0, 6, 24, and 96 h after treatment. The results showed that the expression of *preSUBPEP3* was significantly increased at 6 h, 24 h, and 96 h, indicating that SUBPEP3 can respond to salt stress (Figure 4A). In order to explore the relationship between preSUBPEP3 protein expression quantity and salt stress, we constructed a green fluorescent protein (GFP) expression vector using the promoter of *preSUBPEP3*, which was cloned from the 2488 bp before the open reading frame of *preSUBPEP3,* and *CaMV35S* was used as a control (Figure 4B). The constructed vectors were separately transformed into an agrobacterium, and the agrobacterium was used to infect the tissue culture seedlings of *P. betulifolia* by vacuum infiltration, and then these seedlings were cultured in the culture medium with 300 mM NaCl. After 3 days, the total proteins were extracted and the GFP protein expression was identified by Western blot using GFP antibody. It was found that the expression of GFP in the preSUBPEP3::GFP transient transformed plants was significantly higher than that in the control group (Figure 4C). These results indicated that the mRNA and protein expression of preSUBPEP3 increased significantly when *P. betulifolia* was exposed to salt stress. Tissue-specific analysis showed that the expression of preSUBPEP3 was significantly higher in the xylem than in other tissue (Figure 3D), so to test whether SUBPEP3 responds to salt stress by exhibiting long-distance movement, a fluorescence-labeled tracer method was used to verify the long-distance movement ability of SUBPEP3. The synthetic SUBPEP3 labeled with FAM fluorescence was added to the medium of rooting tissue culture seedlings of *P. betulifolia* (Figure S1A,B). After 3 days, the fluorescence signals of FAM-SUBPEP3 were observed in the roots and leaves of *P. betulifolia* using a laser scanning confocal microscope (LSCM) (Figure 4D). This indicates that SUBPEP3 can move long distances in *P. betulifolia*. Then, the expression of preSUBPEP3 in roots of *P. betulifolia* under salt stress was detected, and the results showed that the expression of preSUBPEP3 was significantly increased in roots at 6 h and 96 h after salt stress (Figure 4E).

### 2.4. SUBPEP3 Could Improve the Salt Tolerance of Pears

**Figure 5:**
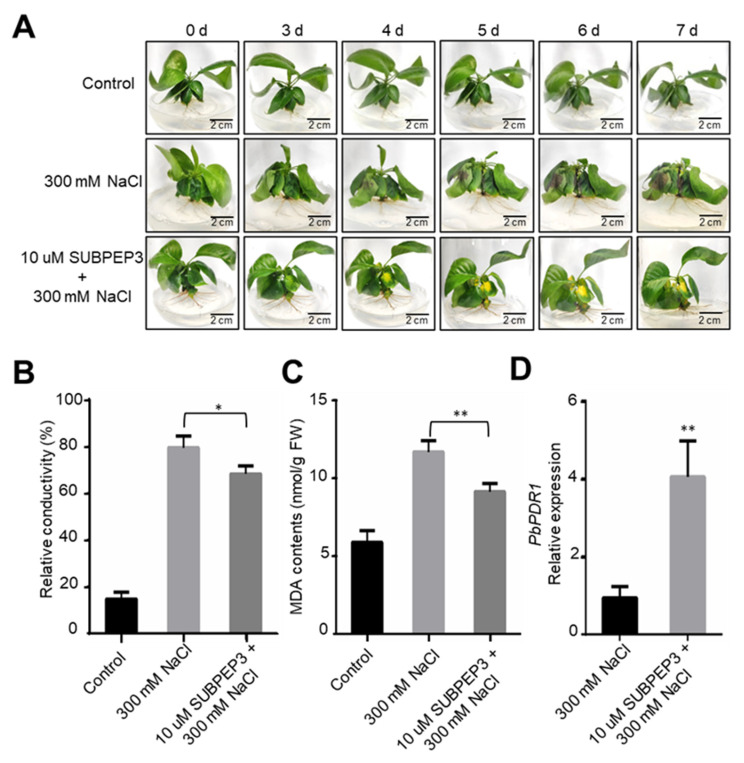
SUBPEP3 improves the salt tolerance of pears. (**A**) Morphology of tissue-cultured *P. betulifolia* in the medium with or without 300 mM NaCl and 10 µM SUBPEP3 at 0, 3, 4, 5, 6, and 7 days after treatment. (**B**,**C**) Relative conductivity (**B**) and malondialdehyde (MDA) contents (**C**) of tissue-cultured *P. betulifolia* at 7 days after treatment. Data are plotted as means ± SD and are based on three biological replicates. * *p* < 0.05 by Student’s *t*-test; ** *p* < 0.01 by Student’s *t*-test. (**D**) Expression of *PbPDR1* in *P. betulifolia* tissue culture seedlings with or without 10 µM SUBPEP3 treatment. Data are plotted as means ± SD and are based on three biological replicates. ** *p* < 0.01 by Student’s *t*-test.

The small secreted peptide SUBPEP3 is derived from the precursor protein preSUBPEP3, and we have found that the expression of the precursor protein preSUBPEP3 increases under salt stress. Therefore, it is hypothesized that SUBPEP3 is related to the response of *P. betulifolia* to salt stress. To investigate the function of SUBPEP3 in the response to salt stress, tissue culture seedlings of the salt-intolerant pear “ZaoJinsu” were cultured in medium containing 300 mM NaCl, and synthetic SUBPEP3 peptide was added to the medium at a final concentration of 10 µM. After treatment, plant phenotypes were observed and physiological parameters were detected. The results showed that the leaves of the control group had obviously wilted after 3 days of salt treatment, but there was no significant change in the plants with SUBPEP3. After 4 days of salt treatment, the leaves of the control plants showed obvious black spots, and after 6 days, the whole plants showed obvious dehydration and wilting. Plants cultured with SUBPEP3 did not show obvious salt damage phenotype under salt treatment, and only a few yellow spots appeared on individual leaves (Figure 5A). After 7 days of salt treatment, the electrical conductivity and malondialdehyde (MDA) content of each group were measured. The results showed that the electrical conductivity and MDA content of the pear with SUBPEP3 treatment were significantly lower than those of the control group without SUBPEP3 (Figure 5B,C), indicating that the cell damage of the pears with SUBPEP3 treatment was significantly lower than that of the control group. And the expression of stress resistance-related gene pleiotropic drug resistance protein 1 (*PbPDR1*) significantly increased, a change which was induced by SUBPEP3 (Figure 5D). These results indicate that SUBPEP3 can improve the salt tolerance of pears. 

### 2.5. Overexpression of SUBPEP3 Enhanced Salt Tolerance

**Figure 6:**
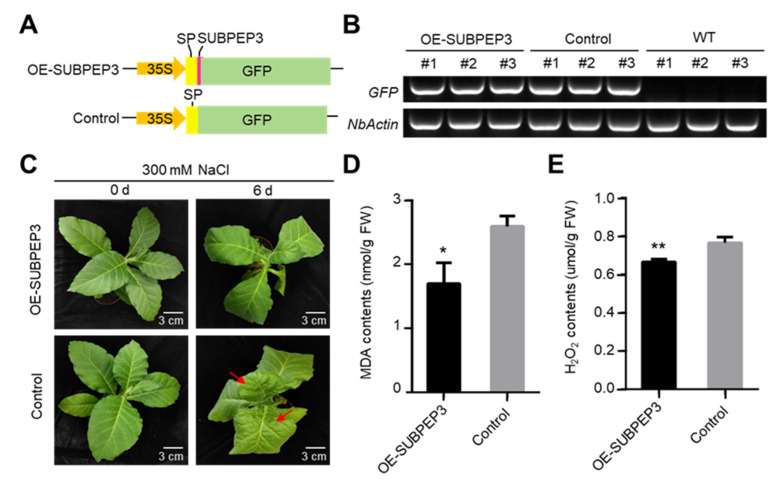
Overexpression of SUBPEP3 enhanced salt tolerance. (**A**) The schematic diagram shows the vectors overexpressing fusion proteins composed by the signal peptide of preSUBPEP3 andGFP, with or without SUBPEP3. SP—signal peptide of preSUBPEP3. (**B**) The RT-PCR analysis of *gfp* expressing level in the *Nicotiana benthamiana* of SUBPEP3-GFP overexpression line (OE- SUBPEP3), control and wild type (WT). *NtActin* was used as the internal control. (**C**) Morphology of transgenic *N. benthamiana* with or without SUBPEP3 overexpression at 7 days after being treated by 300 mM NaCl. (**D**,**E**) MDA contents (**D**) and H_2_O_2_ contents (**E**) of transgenic *N. benthamiana* at 7 days after salt treatment. Data are plotted as means ± SD and are based on three biological replicates. * *p* < 0.05 by Student’s *t*-test, ** *p* < 0.01 by Student’s *t*-test.

To further explore the role of SUBPEP3 in enhancing salt tolerance, we overexpressed SUBPEP3 in *Nicotiana benthamiana*. Firstly, a plant expression vector was constructed with the signal peptide of preSUBPEP3 fused to SUBPEP3, and the vector without SUBPEP3 was used as the control (Figure 6A). SUBPEP3 was overexpressed in *N. benthamiana* by agrobacterium-mediated transformation (Figure 6B). The transgenic tobaccos were then treated with 300 mM NaCl. After 6 days of salt treatment, it was observed that the *N. benthamiana* leaves in the control group showed an obvious state of water loss and shrinkage, while the *N. benthamiana* leaves with SUBPEP3 overexpression only showed a slight tip drooping (Figure 6C). The results showed that the MDA contents and H_2_O_2_ contents of *N. benthamiana* overexpressing SUBPEP3 were significantly lower than those of tobacco in the control group after salt treatment (Figure 6D,E). Based on the above results, it can be concluded that overexpression of SUBPEP3 can significantly improve the salt tolerance of plants. 

## 3. Discussion

*P. betulifolia* has strong tolerance to salt, drought, and other abiotic stresses, so using it as stock to graft different varieties of pears in agricultural production can improve grafted plants’ overall resistance to abiotic stresses [2,22,23]. Therefore, studying the resistance genes in *P. betulifolia* will help to improve the understanding of the molecular mechanism of plants’ resistance to abiotic stress.

Because there are few studies on SSP genes and a large number of SSP genes have not been annotated in the genomes of different species, the annotation of SSP genes is particularly important. In this study, we predicted the SSP genes in the genome of *P. betulifolia*, and a total of 1195 SSP genes were obtained by bioinformatics analysis (Figure 1A and Table S1). A total of 4439 SSP genes of *Medicago truncatula* were predicted in the MtSSPdb database [18]. Using LC-MS/MS to detect conventional peptides in maize and Arabidopsis, a total of 844 conventional peptides were detected in maize and 2363 conventional peptides were detected in Arabidopsis [24]. This indicates that a large number of SSP genes are present in different species, implying that SSPs play an important role in plants. The number of SSPs varies among different species, depending on the quality of the genome, the method of bioinformatics analysis, and the detection conditions of mass spectrometry. Although the SSP precursor proteins in previous studies were found to be small, with most of the SSP precursor proteins being shorter than 250 aa [18], the analysis of the predicted SSP genes in *P. betulifolia* showed that there were still some SSP genes longer than 250 aa, and the length of preSUBPEP3 reached up to 747 aa (Figure 1B). In some previous studies, 250 aa was used as a threshold to screen SSP genes. However, there are still some reports showing that some SSP precursor proteins are longer than 250 aa [25]. This indicates that setting the threshold at 250 aa may lead to some important SSPs being missed. Therefore, we used high homology to known SSP genes as a threshold rather than length.

Functional analysis of SSP genes in *P. betulifolia* showed that the number of SSP genes found to function as signal molecules is the largest, at 57% (Figure 1C). And the research on the function of SSP is mostly carried out around the signal molecules [26]. There were 21 CLAVATA3 (CLV3)/EMBRYO SURROUNDING REGION (ESR)-RELATED (CLE) family genes in *P. betulifolia* (Figure 1D). This is currently the most studied SSP gene family in the genus *Arabidopsis*. It has been shown that CLE is related to the regulation of stem cell proliferation and differentiation in the meristems, embryo and endosperm development, vascular development, and autoregulation of nodulation [27]. Some SSP families with known functions were also found among *P. betulifolia* SSP gene families (Figure 1D and Table S1), such as CEP regulating root development in response to N deficiency signals [15,28]. PLANT PEPTIDE CONTAINING SULFATED TYROSINE (PSY) promotes cell proliferation and expansion [29]. Phytosulfokine (PSK) is a 5-aa tyrosine sulfated peptide that mainly promotes cell proliferation [30,31]. Casparian strip integrity factor 1 (CIF) peptides function to promote the accumulation of hydrophobic substances such as lignin and can regulate the formation of the Casparian band and embryonic cuticle. The function of CIF peptide is closely related to its spatial distribution. And CIF is required to interact with SCHENGEN3/GASSHO1 (SGN3/GSO1) and GSO2 to function [32,33,34]. INFLORESCENCE DEFICIENT IN ABSCISSION (IDA) is a peptide signal for Arabidopsis flower organ shedding [35]. CTLA is the most common gene family in *P. betulifolia* (Figure 1D). Previous studies have only shown that CTLA belongs to the group of peptidase inhibitors, and can inhibit protease activity [36]. But its specific function and mechanism need to be further studied. This indicates that there are differences in SSP genes among different species, and there are still a large number of SSP genes with unknown functions that need to be studied further. Thus, it can be seen that SSP genes in *P. betulifolia* play an important role in various important life activities such as response to external stimuli, signal transduction, regulation of cell differentiation, and tissue development. It involves all stages of plant growth and development, including vegetative and reproductive growth. Therefore, it is of great significance to further verify the gene function and reveal the mechanism of action of SSP genes in *P. betulifolia*. 

The salt-tolerance-related small secreted peptides SUBPEP3 in this study are derived from the PA region of the precursor protein preSUBPEP3 (Figure 3A). preSUBPEP3 belongs to the subtilisin-like protease family (Figure 1D). There are 67 SSP genes belonging to this gene family in *P. betulifolia*, and the large number also shows the importance of this gene family. Previous studies have found that there was a 12aa plant defense peptide signal Glycine max Subtilase Peptide (GmSubPep) derived from the PA domain of the subtilisin-like protease in the leaves of Glycine max. The transcription of GmSubPep was not induced by wounding, methyl salicylate, methyl jasmonate, or ethephon. However, when supplied to soybean suspension cell cultures, GmSubPep peptide can induce the expression of defense-related genes [25]. Phylogenetic analysis of preSUBPEP3 and SUBPEP3 revealed that they are conserved in different species (Figure 3B,C). This implies that SUBPEP3 is also highly likely to be a signaling molecule in response to external stimuli. In this study, we found that the expression of preSUBPEP3 could be induced by salt stress (Figure 4A,C), and the addition to pear culture medium (Figure 5A–D) and overexpression in tobacco (Figure 6A–E) of SUBPEP3 could significantly improve the salt tolerance of plants. As in whole plantlets, preSUBPEP3 expression was increased in roots under salt stress (Figure 4E). Furthermore, our results showed that SUBPEP3 could move long distances from roots to leaves (Figure 4D). Therefore, we hypothesized that the expression of preSUBPEP3 in roots was increased under salt stress, leading to the production of a large number of SUBPEP3 that moved to the leaves over a long distance to transmit the signal of salt stress, thereby causing a downstream cascade to improve the salt tolerance of the plant. The specific function mechanism is in need of further study. 

As reported in previous studies, SSPs usually function by binding to the leucine-rich-repeat receptor-like kinase (LRR-RLK) on the cell membrane [37]. Studies have been conducted in several different ways to find receptors that correspond to SSPs. One way is the photoaffinity-labeling method, in which the proteins overexpressed by the expression library of *Arabidopsis* LRR-RLKs subfamily X and XI in tobacco BY-2 cells were incubated with photoaffinity-labeled peptides to verify their interactions [14]. Several ligand–receptor pairs were identified by this approach, including the GASSHO 1/SCHENGEN 3 receptor, which directly interacts with CIF1 and CIF2 peptides [33]; the C-TERMINALLY ENCODED PEPTIDEs (CEPs) and CEP RECEPTOR (CEPR) interaction [15]; and BARELY ANY MERISTEM 1 (BAM1), which interacts with CLV3 [38]. Structural biology can also help predict ligand–receptor pairs. The root meristem growth factor (RGF) receptor RGFR1 was identified by purifying the extracellular LRR domain of LRR-RLKs, incubating with chemically synthesized peptides, and combining gel filtration and mass spectrometry [39]. Another method involves detecting peptide-induced changes in plasma membrane protein phosphorylation through quantitative phosphoproteomics. Synthetic RALF1 was used to induce changes in the phosphorylation level of the target protein and screen out the receptor protein FER [40]. The action mechanism of SUBPEP3 in inducing plant salt tolerance and the binding of SUBPEP3 to its receptor represent potential research topics for future studies.

At present, the method of exogenously applying synthetic SSPs to plants or plant cells has been widely used to verify the function of SSPs [16,25]. Plant hormones such as auxin and gibberellins, which are also signal molecules like SSPs, have been widely used as plant growth regulators in agricultural production [41]. In the future, after further optimization which will involve improving the chemical production process to reduce the production cost of synthetic SSPs, and improving its stability and ability to enter plant cells by combining with nanomaterials, synthetic SSPs is expected to be applied to regulate plant growth and development in agricultural production.

In summary, we annotated SSP genes in the genome of *P. betulifolia*, which improved the understanding of the characteristics of SSP genes in Rosaceae fruit trees and laid a theoretical foundation for further studies on the function and the action mechanism of SSPs. Based on this, our study identified a small secreted peptide, SUBPEP3, which was found to enhance plant salt tolerance. This finding has great significance for the application of SSPs in the regulation of plant stress resistance, as well as the cultivation of superior stress-resistant varieties.

## 4. Materials and Methods

### 4.1. Plant Materials and Growth Conditions

*P. betulifolia* seedlings were grown in soil at 22 °C with supplemental lighting (16 h light:8 h dark) in a greenhouse at China Agricultural University, Beijing, China. *P. betulifolia* tissue culture plantlets were cultured on Murashige & Skoog (MS) (phyto technology) medium containing 1 mg/L 6-benzylaminopurine (6-BA) and 0.5 mg/L 1-Naphthaleneacetic acid (NAA) (Sangon Biotech, Shanghai, China). All *P. betulifolia* plants were derived from the Shangzhuang experimental station of China Agricultural University, Beijing, China. *Nicotiana tabacum* (*Nicotiana tabacum* var. Wisconsin 38) tissue culture plantlets were cultured on Murashige and Skoog (MS) medium. All the cultivation medium contained 30 g/L sucrose and 7 g/L agar (Sinopharm, Beijing, China). The caps of tissue culture bottles are rigid with membranes. The environmental conditions for the growth of the tissue culture plantlets were 16 h light, 8 h dark, and 22 °C under a 250 µmol/m^2^/s illumination intensity provided by cool-white fluorescent tubes and a relative humidity of 80%. All tissue culture plantlets were cultured in the tissue culture room of China Agricultural University, Beijing, China.

### 4.2. SSP Genes Annotation

The *P. betulifolia* genome was downloaded from GDR (https://www.rosaceae.org/, accessed on 5 July 2023). The signal peptide domains were predicted by SignalP 5.0 (https://services.healthtech.dtu.dk/services/SignalP-5.0/, accessed on 5 July 2023) [42]. The transmembrane domains analysis was conducted by TransMembrane prediction using Hidden Markov Models (TMHMM) (https://services.healthtech.dtu.dk/services/TMHMM-2.0/, accessed on 5 July 2023) [43]. The hidden Markov models (HMMs) were applied to search for SSP gene homologs in the genome of *P. betulifolia* performed by SSP-Prediction (https://mtsspdb.zhaolab.org/prediction/, accessed on 5 July 2023) [18]. Finally, genes of proteins with N-terminal signal peptides, no transmembrane domains, and high homology to known SSP proteins were annotated as SSP genes.

### 4.3. GO and KEGG Enrichment Analysis

The Gene ontology (GO) and Kyoto Encyclopedia of Genes and Genomes (KEGG) annotation were performed by eggNOG-Mapper (http://eggnog-mapper.embl.de/, accessed on 4 August 2023) [44]. The GO and KEGG enrichment analysis were conducted by TBtools-II v2.012 [45].

### 4.4. Domain Prediction and Phylogenetic Analysis

The conserved domains of sequences were predicted by NCBI CD-search (https://www.ncbi.nlm.nih.gov/Structure/cdd/wrpsb.cgi/, accessed on 4 August 2023). Phylogenetic analysis was performed by Molecular Evolutionary Genetics Analysis 5.0 (MEGA5) [46].

### 4.5. RNA Extraction and qRT-PCR

The samples subjected to RNA extraction were stored at −80 °C. Before RNA extraction, samples were ground under liquid nitrogen conditions. The total RNA of *P. betulifolia* was extracted by the cetyltrimethyl ammonium bromide (CTAB) method [47]. The total RNA of tobacco was extracted by TRIzol reagent (Invitrogen). Extracted RNA was treated with DNase I (Promega, Madison, WI, USA) for 40 min at 37 °C to avoid genomic DNA contamination. Total RNA was reverse transcribed into cDNA by TRUEscript RT MasterMix (Aidlab Biotechnologies) according to the product manuals. The cDNA for qRT-PCR was diluted 10 times. And an qRT-PCR assay was carried out on Thermo Fisher Scientific’s stepone Plus real-time PCR instrument with SuperReal premix Plus (TIANGEN BIOTECH (BEIJING), Beijing, China). For qRT-PCR analysis, 2 μL diluted DNA was used as template in a reaction under the following conditions: 95 °C for 15 min, and 40 cycles of 95 °C for 10 s and 60 °C for 32 s. The fold changes in target gene expression were normalized using *PbACTIN* as the internal control and evaluated by the 2^−ΔΔ*C*t^ method. All primers used are listed in Table S3. All assays were based on three biological replicates.

### 4.6. Peptide Synthesis

Synthetic peptides were synthesized by Sangon Biotech (Shanghai) with the purity > 95%.

### 4.7. Plasmid Construction

The promoter of *preSUBPEP3* was cloned from the 2488 bp before the open reading frame of *preSUBPEP3*. The fragments replaced the CaMV35S promoter of pCAMBIA2300 to generate preSUBPEP3pro::GFP using MfeI and BamHI restriction sites. The fragments of the signal peptide of preSUBPEP3 and SUBPEP3 were inserted into pCAMBIA2300 using KpnI and BamHI restriction sites to generate pSUBPEP3-GFP. The same plasmid without SUBPEP3 was used as the control. Plasmids were constructed by a ClonExpress II One Step Cloning Kit (Vazyme, Nanjing, China) according to the product manuals. All constructs were confirmed by sequencing and then transformed into *Agrobacterium tumefaciens* strain GV3101.

### 4.8. Transient Expression and Plant Transformation

The agrobacterium cultured overnight was resuspended in suspension buffer (10 mM MgCl_2_, 10 mM MES (pH 5.6), and 0.1 mM acetosyringone) with the absorbance value of 1.0 at a wavelength of 600 nm (OD_600_ = 1.0). After being kept at room temperature for 2 to 4 h, the bacterial suspension infected the *P. betulifolia* tissue culture seedlings by vacuum infiltration. And then the seedlings were cultured for 1 d in the dark and 2 d in the normal light conditions.

*N. tabacum* was used as the genetic background of the transgenic lines. And the transform of *N. tabacum* was performed by leaf disc transformation [48].

### 4.9. Western Blot

Total protein of *P. betulifolia* tissue culture seedlings was extracted by the Plant Total Protein Extraction Kit (HuaXingBoChuang Bio, Beijing, China) and separated by SDS-PAGE. The primary antibody was GFP-tag mouse monoclonal antibody that was used at 1:3000 (*v*/*v*) dilution. The secondary antibody was horseradish peroxidase-conjugated goat anti-mouse immunoglobulin G that was used at 1:10,000 (*v*/*v*) dilution.

### 4.10. Salt Treatment and Salt Tolerance Assay

Plantlets planted in soil were watered with 300 mM NaCl. Tissue culture seedlings were grown in medium with 300 mM NaCl. The leaves of the plantlets were collected for physiological index measurement, including electrolyte leakage, MDA, and H_2_O_2_ contents. The electrolyte leakage, MDA, and H_2_O_2_ contents of plant leaves were measured as previously reported [49,50,51].

### 4.11. Subcellular Localization

The fragments of the signal peptide of *preSUBPEP3* and *SUBPEP3* were inserted into pCAMBIA2300 using KpnI and BamHI restriction sites to generate pSUBPEP3-GFP. The vectors were transformed into *A. tumefaciens* strain GV3101 and were infiltrated in *N. benthamiana* leaves. Three days after infiltration, GFP fluorescence was detected using a Zeiss LSM900 confocal microscope at 488 nm excitation light wavelength.

## Data Availability

The data presented in this study are available in the article or in the Supplementary Materials.

## Supplementary Materials

The following supporting information can be downloaded at https://www.mdpi.com/article/10.3390/ijms25094612/s1.
