# Supplementary material for: Unraveling a Small Secreted Peptide SUBPEP3 That Positively Regulates Salt-Stress Tolerance in Pyrus betulifolia
Source: Int J Mol Sci. 2024 Apr 23;25(9):4612. doi: 10.3390/ijms25094612 (PMC11083645; doi:10.3390/ijms25094612)
Supplement: Supplementary file 1 [file ijms-25-04612-s001.zip › Figure S1.pdf]

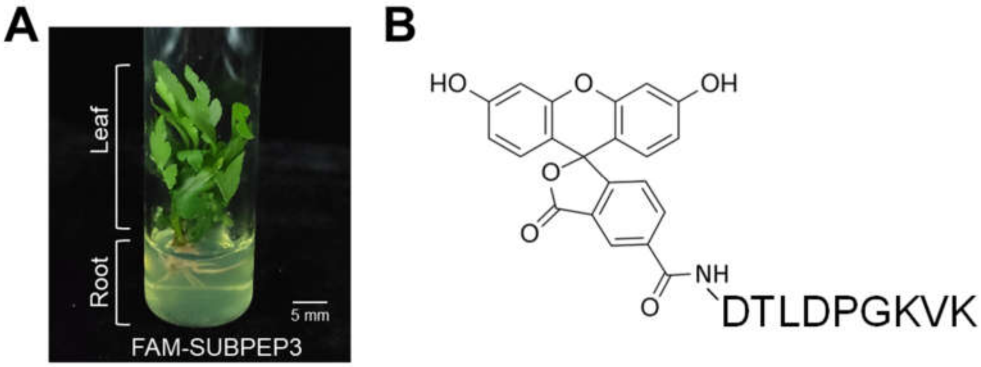

Figure S1. The mobility identification of SUBPEP3 in *P. betulaefolia*. (**A**) The photograph of *P. betulaefolia* culture seedlings cultured in the medium with 10 μM FAM-SUBPEP3. (**B**) Structure of FAM-SUBPEP3.

Table S1. The SSP genes identified in the *Pyrus betulaefolia* genome.

Table S2. The gene function annotation, GO and KEGG analysis of SSP genes identified in the *Pyrus betulaefolia* genome.

Table S3. Primers used in this study.
